# Supplementary material for: Enhanced Performance of WO3/SnO2 Nanocomposite Electrodes with Redox-Active Electrolytes for Supercapacitors
Source: Int J Mol Sci. 2023 Mar 23;24(7):6045. doi: 10.3390/ijms24076045 (PMC10094020; doi:10.3390/ijms24076045)
Supplement: Supplementary file 1 [file ijms-24-06045-s001.zip › ijms-2241113-supplementary.pdf]

Supporting information

Enhanced Performance of WO_3/SnO_2 Nanocomposite Electrodes with Redox-Active Electrolytes for Supercapacitors

Tamiru Deressa Morka and Masaki Ujihara*

Graduate Institute of Applied Science and Technology, National Taiwan University of Science and Technology,43 Keelung Road, Taipei 10607, Taiwan

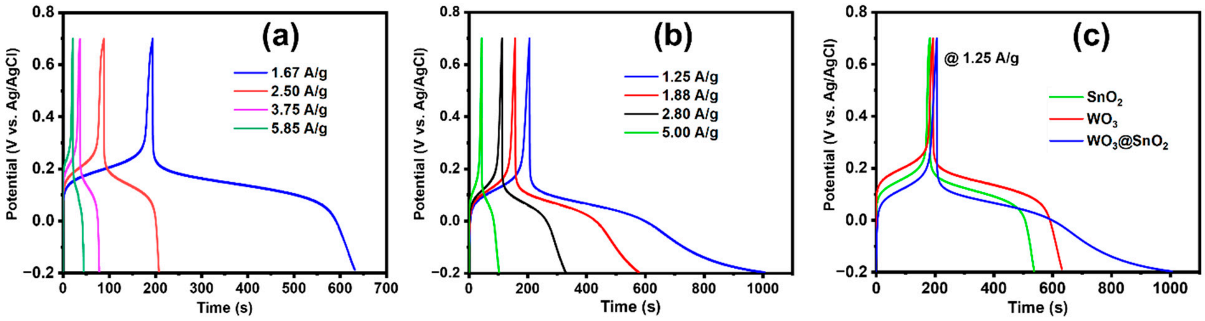

Fig. S1 GCD curves of (a) WO_3 electrode and (b) WO_3/SnO_2 electrode at various current density in 1M Na_2SO_4/0.02 M $K_3Fe(CN)_6$ electrolyte; and (c) comparison of WO_3, SnO_2, and WO_3/SnO_2 at same current density.
